# Prospective, Multi-Institutional Observational Study of Deterioration in Activities of Daily Living in Elderly Patients After Lung Cancer Surgery

**DOI:** 10.1016/j.jtocrr.2023.100550

**Published:** 2023-07-17

**Authors:** Hidefumi Takei, Hideo Kunitoh, Masashi Wakabayashi, Tomoko Kataoka, Yuta Sekino, Tomonori Mizutani, Masahiro Tsuboi, Norihiko Ikeda, Hisao Asamura, Morihito Okada, Makoto Takahama, Yasuhisa Ohde, Jiro Okami, Satoshi Shiono, Keijyu Aokage, Shun-ichi Watanabe, Lung Cancer Surgical Study Group of the Japan Clinical Oncology Group, Japan, H. Fukuda, T. Shibata

**Affiliations:** a Department of Thoracic Surgery, Kyorin University, Tokyo, Japan; b Department of Thoracic Surgery, Showa University, Tokyo, Japan; c Department of Medical Oncology, Japanese Red Cross Medical Center, Tokyo, Japan; d Japan Clinical Oncology Group Data Center/Operations Office, National Cancer Center Hospital, Tokyo, Japan; e Department of Medical Oncology, Kyorin University, Tokyo, Japan; f Department of Thoracic Surgery, National Cancer Center Hospital East, Kashiwa, Japan; g Department of Surgery, Tokyo Medical University, Tokyo, Japan; h Division of Thoracic Surgery, Keio University, Tokyo, Japan; i Department of Surgical Oncology, Hiroshima University Hospital, Hiroshima, Japan; j Department of General Thoracic Surgery, Osaka City General Hospital, Osaka, Japan; k Department of Thoracic Surgery, Shizuoka Cancer Center, Shizuoka, Japan; l Department of General Thoracic Surgery, Osaka International Cancer Institute, Osaka, Japan; m Department of Thoracic Surgery, Yamagata Prefectural Central Hospital, Yamagata, Japan; n Department of Surgery II, Faculty of Medicine, Yamagata University Hospital, Yamagata, Japan; o Department of Thoracic Surgery, National Cancer Center Hospital, Tokyo, Japan

**Keywords:** Non–small cell, surgery, Elderly patients, Activities of daily living, Quality of life

## Abstract

**Introduction:**

To determine the rate of deteriorating activities of daily living (ADL) and to investigate predictive factors in elderly patients undergoing surgery for NSCLC.

**Methods:**

Patients with NSCLC aged 75 years or older who underwent curative surgical resection were evaluated using the Tokyo Metropolitan Institute of Gerontology Index of Competence Instrumental ADL (TMIG-IADL) and the Japanese version of EuroQol 5-dimensions 5-level (EQ-5D-5L) quality-of-life scale administered at baseline and at 6 months postoperative. The primary end point was the rate of living patients without substantial deterioration of TMIG-IADL, defined as a decline greater than or equal to three points. Multivariable logistic regression was performed to determine risk factors for deteriorating ADL.

**Results:**

Between May 2019 and May 2020, 876 of the 986 screened patients enrolled from 47 institutions were eligible and included in the analysis. TMIG-IADL and EQ-5D-5L scores were obtained from 96.0% and 92.6% of the patients, respectively. At 6 months postoperative, 745 patients (85.1%, 95% confidence interval: 82.5%–87.3%) reported no significant ADL deterioration, and 96 of 841 patients (11.4%) with postoperative score data reported significant deterioration. The social domain was the most frequently affected activity. In multivariable analysis, poor performance status, low G8 geriatric screening score, segmentectomy (versus wedge resection), and surgery lasting less than 3 hours were associated with deteriorating ADL. Worsening EQ-5D-5L scores by minimally important difference or more were observed in 22.1% of the patients. Changes in TMIG-IADL and EQ-5D-5L scores were poorly correlated.

**Conclusions:**

Approximately 15% of elderly patients with NSCLC experienced significant ADL deterioration at 6 months postoperative.

## Introduction

Lung cancer is a leading cause of cancer-related deaths in Japan, with more than 75,000 deaths reported in 2020. With the aging of the general population, the incidence of lung cancer is expected to increase, with more elderly patients undergoing surgical resection. According to a survey by the Japanese Society of Thoracic Surgery,^1^ 44,859 patients in Japan underwent surgery for primary lung cancer in 2018 and nearly 60% of these patients were aged 70 years or older. In fact, 6115 of the patients who underwent resection (14%) were 80 years or older, and both the number and proportion continue to increase.

Many studies have investigated the postoperative outcomes of elderly patients with lung cancer. A Japanese study, which included 367 patients aged 80 years and older who underwent surgical resection for clinical stage I lung cancer^2^ reported that serious complications occurred in 8.4% of the patients and that the rates of postoperative mortality and 5-year survival were 1.4% and 55.7%, respectively. A recent prospective cohort study by the Japanese Association for Chest Surgery^3^ evaluated 895 octogenarians using a comprehensive scoring system for surgical risk and reported a 30-day postoperative mortality rate of 1.0% and a 3-year survival rate of 86.7%. This study^3^ also identified several predictive factors for surgical risk and survival. However, most of the previous studies on elderly patients with NSCLC only evaluated postoperative morbidity and mortality^4^^,^^5^ and overall survival (OS), and not functional outcomes.

Postoperative activities of daily living (ADL) are critically important for both patients and their families. Although the importance of functional outcomes in elderly populations has been reported,^6^, ^7^, ^8^, ^9^, ^10^ few studies have evaluated postoperative ADL and quality of life (QOL) despite the notable impact of surgical stress on frailty in elderly patients.^5^^,^^11^^,^^12^

Whereas the prognosis of patients with lung cancer undergoing surgery has markedly improved, with 5-year OS rates of 90% or more for node-negative NSCLC, comorbidities including second primary cancers account for a significant portion of deaths.^13^, ^14^, ^15^ Treatment for these comorbidities can be compromised by frailty owing to postoperative deterioration of the patient’s physical condition.^16^

In elderly patients with lung cancer, therefore, ADL and QOL after surgery are important not only for their care and comfort but also for their prognosis. Without the information on postoperative ADL and QOL, patients and families cannot make well-informed choices for treatment among available options including surgery, radiotherapy, chemotherapy, immunotherapy, and supportive care.^10^^,^^17^

We conducted an observational study to evaluate postoperative ADL and QOL in elderly patients aged 75 years and older with NSCLC. We aimed to elucidate the rate of ADL deterioration in elderly patients undergoing surgical treatment for NSCLC and to determine the predictive factors for ADL deterioration. We used the Japanese version of EuroQol 5-dimensions 5-level (EQ-5D-5L),^18^^,^^19^ a globally validated QOL assessment tool, and the Tokyo Metropolitan Institute of Gerontology Index of Competence Instrumental ADL (TMIG-IADL),^20^^,^^21^ a 13-item validated scale for geriatric ADL and is the only scale of its kind available in Japan.

Here we report the ADL and QOL of patients at 6 months postoperatively, which are the main objectives of the study.

## Materials and Methods

### Eligibility

**Figure 1:**
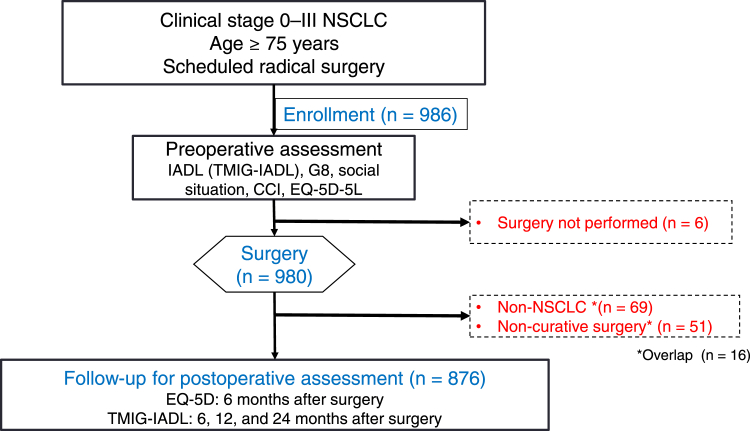
Study flow chart. CCI, Charlson comorbidity index; EQ-5D, EuroQol 5-dimensions; G8, geriatric screening tool; IADL, instrumental activities of daily living; TMIG-IADL, Tokyo Metropolitan Institute of Gerontology Index of Competence Instrumental Activities of Daily Living.

Figure 1 summarizes the study flow. Patients aged 75 years or older with radiologically suspected clinical stage 0 to III NSCLC were enrolled. Patients with tumors that were determined as amenable to complete resection and were planned to undergo primary surgery were included. The other inclusion criteria were as follows: (1) radical surgery scheduled within 14 days after enrollment; (2) competency to undergo comprehensive geriatric assessments using TMIG-IALD, G8 geriatric screening, Charlson comorbidity index (CCI), and engaged in social situations before surgery; and (3) competency to communicate in written Japanese. Pathologic diagnosis before surgery was not mandatory. Patients with active invasive malignancy with a disease-free period of shorter than 5 years, those with induction treatment or previous chemotherapy, and those with a history of previous surgical lung resection were excluded.

All patients provided written informed consent form before study enrollment. The present study was conducted according to the tenets of the Declaration of Helsinki and approved by the institutional review boards of all participating institutes (Japanese Red Cross Medical Center institutional review board approval number: 2019-973, on April 26, 2019). The study was registered with the UMIN Clinical Trials Registry (UMIN000036796).

### Study Design and Treatment

This was a prospective, multi-institutional observational study conducted by the Lung Cancer Surgical Study Group of Japan Clinical Oncology Group.

Baseline geriatric function assessment before surgery was performed using TMIG-IADL^20^^,^^21^ (see Supplementary Table 1*A* for specific questionnaires and scores) and G8^22^ (see Supplementary Table 1*B* for specific questionnaires and scores). Baseline information on the social situation, such as habitation, CCI score^23^ (see Supplementary Table 1*C* for specific conditions and scores), polypharmacy status,^24^ and Japanese version EQ-5D-5L scores^18^^,^^19^^,^^25^ (see Supplementary Table 1*D* for specific questionnaires), were also obtained before surgery. Surgical procedures were not specified in the study protocol. Patients with a final pathologic diagnosis of NSCLC were observed for ADL outcomes. Patients undergoing noncurative surgery and those with neuroendocrine tumors, such as small cell carcinoma, large cell neuroendocrine carcinoma, or carcinoid tumor were excluded from follow-up and subsequent data acquisition.

The EQ-5D-5L questionnaire was administered at 6 months, with responses directly mailed to the research office by the patients, whereas the TMIG-IADL questionnaire was administered by attending physicians at 6, 12, and 24 months to evaluate the long-term surgical impact on ADL. The present study reports the data at postoperative 6 months, which include the primary end point described below.

### End Points

The primary end point was the rate of patients without ADL deterioration at 6 months after surgery. The TMIG-IADL^20^^,^^21^ (Supplementary Table 1*A*) is a 13-item index of competence on three domains and includes five items in instrumental self-maintenance, four items in effectance (defined as effective interaction with one’s environment) or intellectual activity, and four items in social role. A higher TMIG-IADL score indicates better capacity for activity. A previous Japanese study^26^ revealed that the SD of TMIG-IADL score was 3.0 points in the elderly population; therefore, in the present study, the protocol-specified ADL deterioration was defined as a decline of at least three points in TMIG-IADL score or missing ADL data, because it was presumed that it is likely that most of the missing data actually indicate worsening of the patient’s condition. This cutoff was on the basis of the distribution-based method to determine the minimally important difference (MID).^27^ A sensitivity analysis with the threshold of “ADL deterioration” with a change in score of two points was also performed; this “criteria-modified” ADL deterioration was defined as TMIG-IADL score deterioration of at least two points or missing data.

Patients with missing postoperative TMIG-IADL data were classified as exhibiting ADL deterioration. The time point of 6 months was determined by consensus of the surgeons on the basis of the consideration that elderly patients were extremely unlikely to achieve functional recovery after 6 months after surgery.

The secondary end points were TMIG-IADL scores at 12 and 24 months, EQ-5D-5L score at 6 months, OS, relapse-free survival (RFS), and rates of serious postoperative complications, defined as greater than or equal to grade 3 within 30 days of surgery according to the Common Terminology Criteria for Adverse Events version 5.0-Japan Clinical Oncology Group criteria.

EQ-5D-5L QOL scores were calculated according to the method by Ikeda et al.^25^ The MID was set at 0.061 for the EQ-5D-5L QOL scores, as reported by Shiroiwa et al.^28^

### Statistical Analysis

The calculated sample size was 810 to obtain the half-width of a 95% confidence interval (CI) for the primary end point of within 3.5%. To account for ineligible patients, the planned sample size was set at 1000. A two-sided *p* value of less than 0.05 was considered statistically significant. The rate of patients without TMIG-IDAL deterioration and the CI were estimated on the basis of binomial distribution. To determine risk factors for ADL deterioration, univariable and multivariable logistic regression analyses were performed using various demographic and clinical variables (see Supplementary Table 1*E* for specific classifications of factors). These variables were used to estimate the rate of patients without ADL deterioration at 6 months. The OS and RFS are estimated using the Kaplan-Meier method.

Exploratory analyses were performed to determine the extent to which the three domains (instrumental self-maintenance, effectance, and social role) were affected in patients with ADL deterioration.

Deterioration of EQ-5D-5L scores by more than the MID and the CI were estimated on the basis of binomial distribution, and the correlation between the changes in EQ-5D-5L and TMIG-IADL scores was evaluated by Spearman’s rank coefficient.

All statistical analyses were performed using SAS release 9.4 (SAS Institute, Cary, NC).

## Results

### Study Cohort

A total of 986 patients from 47 institutions were enrolled between May 20, 2019 and May 29, 2020. According to the final pathologic results, 876 patients had NSCLC, underwent complete resection, and were followed up to assess ADL and QOL (Fig. 1).

Table 1 details the study cohort characteristics. A total of 301 (34.4%) and 71 patients (8.1%) were aged 80 to 84 years and 85 years old and above, respectively. The remaining 504 patients (57.5%) were aged 75 to 79 years. Only three patients (0.3%) underwent pneumonectomy. Combined resection, such as pulmonary arterioplasty, bronchoplasty, and costal resection, was performed in 13 patients (1.5%).

**Table 1:** Baseline Characteristics and Surgical and Pathologic Factors of 876 Patients With NSCLC Who Underwent Complete Resection

Characteristics	Number of Patients (%)					
Sex						
Male	491 (56.1)					
Female	385 (43.9)					
Age (y)		Median: 79	Range: 75–92	Q1–Q3: 77–82		
Clinical stage (UICC-TNM eighth classification)						
0	29 (3.3)					
IA1	131 (15.0)					
IA2	254 (29.0)					
IA3	158 (18.0)					
IB	134 (15.3)					
IIA	36 (4.1)					
IIB	96 (11.0)					
IIIA	35 (4.0)					
IIIB	3 (0.3)					
ECOG Performance status						
0	717 (81.8)					
1	152 (17.4)					
2	5 (0.6)					
3	1 (0.1)					
4	1 (0.1)					
Smoking history						
Never	363 (41.4)					
Ever	513 (58.6)					
Smoking years		Median: 43	Range: 0–66	Q1–Q3: 30–54		
Number of daily cigarettes		Median: 20	Range: 0–80	Q1–Q3: 15–30		
Emphysema						
No	623 (71.1)					
Yes	253 (28.9)					
Interstitial pneumonia						
No	788 (90.0)					
Yes	88 (10.0)					
Number of medications						
0	63 (7.2)					
1-3	255 (29.1)					
4-9	464 (53.0)					
≥ 10	94 (10.7)					
Operation						
Wedge Resection	95 (10.8)					
Segmentectomy	133 (15.2)					
Lobectomy	639 (72.9)					
Bilobectomy	6 (0.7)					
Pneumonectomy	3 (0.3)					
Lymph node dissection						
ND0–1	371 (42.4)					
ND2a or more	505 (57.6)					
Combined resection						
No	863 (98.5)					
Yes	13 (1.5)					
Operation time (min)		Median: 157	Range: 33–524	Q1–Q3: 119–205		
Histology						
Adenocarcinoma	678 (77.4)					
Squamous cell carcinoma	169 (19.3)					
Large cell carcinoma	1 (0.1)					
Adenosquamous carcinoma	16 (1.8)					
Others	12 (1.4)					
Pathologic stage (UICC-TNM Eighth classification)						
0	31 (3.5)					
IA1	171 (19.5)					
IA2	207 (23.6)					
IA3	114 (13.0)					
IB	127 (14.5)					
IIA	45 (5.1)					
IIB	96 (11.0)					
IIIA	74 (8.4)					
IIIB	11 (1.3)					
Postoperative chemotherapy						
None	837 (95.5)					
Tegafur-uracil	30 (3.4)					
Platinum-based	8 (0.9)					
Missing	1 (0.1)					
Postoperative radiotherapy						
None	874 (99.8)					
Yes	1 (0.1)					
Missing	1 (0.1)					
G8 score at enrollment		Mean: 14.0	Median: 14	SD: 1.8	Range: 6.5–17	
CCI score		Mean: 0.9	Median: 0	SD: 1.2	Range: 0–8	
Baseline TMIG-IADL score		Mean: 11.6	Median: 12	SD: 1.8	Range: 2–13	Q1–Q3: 11–13

CCI, Charlson comorbidity index; ECOG, Eastern Cooperative Oncology Group; G8, geriatric screening tool; ND0, no node dissection; ND1, hilar node dissection; ND2, mediastinal node dissection; TMIG-IADL, Tokyo Metropolitan Institute of Gerontology Index of Competence Instrumental Activities of Daily Living; Q1, first quartile; Q3, third quartile; UICC, Union for International Cancer Control.

The mean plus or minus SD of the baseline TMIG-IADL scores were 11.6 plus or minus 1.8. The SD was smaller than that previously reported in the general population, implying the study population, selected for surgical treatment, might be less heterogeneous in terms of ADL.

### Survival and Postoperative Complications

During a median follow-up of 6.2 months, 14 of the 870 patients with available follow-up data died; the causes were primary lung cancer and other causes in 4 and 10 cases, respectively. Among 868 patients with data on relapse and follow-up, 44 patients died or experienced a relapse. The 6-month OS and RFS rates were 98.7% and 96.0%, respectively.

Grade 3 or higher postoperative complications occurred in 86 of the 876 patients (9.8%) who underwent complete resection. The most frequent complication was pulmonary leakage (3.1%) followed by lung infection (1.4%). Grade 4 postoperative complications occurred in 19 patients (2.2%). There were three deaths (0.3%) during the 30-day postoperative period.

### The TMIG-IADL Data

**Figure 2:**
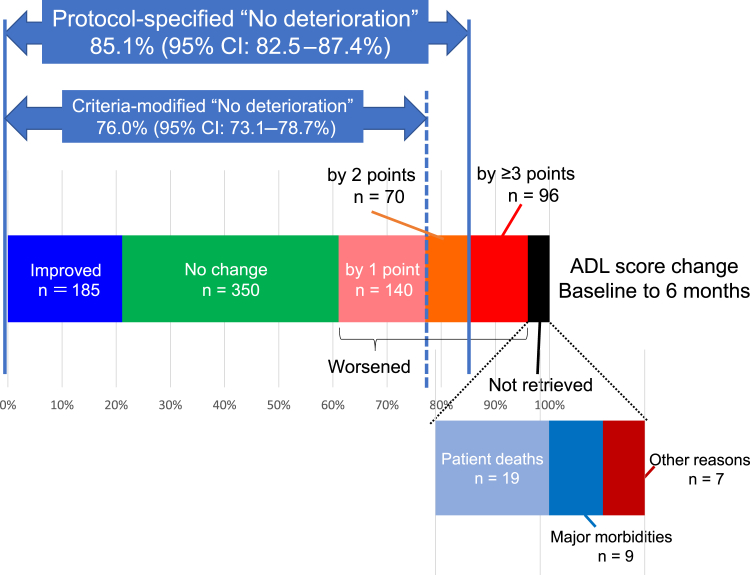
Changes of the TMIG-IADL (Tokyo Metropolitan Institute of Gerontology Index of Competence Instrumental Activities of Daily Living) scores from baseline to postoperative 6 months. Colored columns represent patients with TMIG-IADL scores improvement, no change, worsening by one point, worsening by two points, worsening by three points or more, or missing postoperatively. Those with missing data are then subclassified according to the reasons: patient deaths, major morbidities and others. The rate of patients without a score worsening by greater than or equal to three points or missing (protocol-specified “no deterioration”) is the primary end point of the study, whereas criteria-modified “no deterioration” means TMIG-IADL score deterioration of greater than or equal to two points or missing data. ADL, activities of daily living; CI, confidence interval.

The TMIG-IADL questionnaire data were not available for 35 patients; the causes were death, incapacitating complications such as brain infarction, and other or unknown reasons in 19, nine, and seven patients, respectively (Fig. 2). Thus, the TMIG-IADL questionnaire data were successfully retrieved from 96.0% (841 of 876) of all patients and 98.1% (841 of 857) of all living patients.

At postoperative 6 months, the TMIG-IADL score changes ranged from −13 to +5; the scores worsened (by one point or more) in 306 (36.4%), stable in 350 (41.6%) and improved in 185 (22.0%), respectively, among the 841 patients for whom the scores were retrieved (Fig. 2). There was a statistically significant trend for worsening of the score (*p* < 0.0001 by Wilcoxon signed rank test), even after excluding dead or incapacitated patients for whom postoperative scores were not available. This trend was observed in almost every strata of sex, age, tumor stage, PS, smoking, operation method, or baseline G8, except for those who underwent wedge resection. A total of 95 patients underwent wedge resection, and their postoperative TMIG-IADL scores were missing/worsened in 31 (32.6%), stable in 41 (43.2%), and improved in 23 (24.2%), without significant overall change (*p* = 0.18 by Wilcoxon signed rank test)

### Patients With and Without ADL Deterioration

In 745 of the 876 patients, the TMIG-IADL scores either improved (in 185 patients or 21.1%), did not change (in 350 patients or 40.0%), or worsened by less than or equal to two points (in 210 patients or 24.0%) at postoperative 6 months. TMIG-IADL score deterioration of greater than or equal to three points was observed in 96 patients (11.0%). The rate of patients without protocol-specified ADL deterioration (missing or worsened by ≥3 points) was 85.1% (95% CI: 82.5%–87.3%) (Fig. 2).

Because the SD of the study population was 1.8, we made a sensitivity analysis with the threshold of “ADL deterioration” changed to two scores. TMIG-IADL score deterioration of greater than or equal to two points was observed in 166 patients (19.7% of the 841 patients), and, including the missing data counted as deterioration, the criteria-modified overall rate of “patients without ADL deterioration” was 76.0% (95% CI: 73.1%–78.7%) (Fig. 2).

The rate of ADL deterioration was higher in patients with grade 3 or higher serious postoperative complications (n = 86) than in those without (n = 790). The rates of patients without ADL deterioration were 61.6% and 87.6% in patients with and without serious postoperative complications, respectively, with an OR of 4.397 (95% CI: 2.711–7.129, *p* < 0.0001) to experience ADL deterioration. Of the 86 patients with grade 3 or higher serious postoperative complications, 21 (24.4%) and 16 (18.6%) reported stable and improved scores, respectively.

### Subset Analysis of Factors Predicting ADL Nondeterioration

The univariable analysis was performed to identify factors associated with ADL nondeterioration at 6 months (Table 2). ADL deterioration was significantly more frequent among male patients, those aged 80 years or older, those with a performance status (PS) (by Eastern Cooperative Oncology Group) score of at least 1, and those with a smoking history of more than 20 years. Patients with emphysema, interstitial pneumonitis, low G8 scores, and high CCI scores were significantly more likely to experience ADL deterioration at 6 months. In addition, those treated with at least four regular medications were more likely to be associated with ADL deterioration at 6 months. There was no clear association of ADL deterioration with surgical procedures including the extent of lung resection; however, patients undergoing combined resection were significantly more likely to experience ADL deterioration.

**Table 2:** Univariable Analyses of Factors Associated With Nondeterioration of ADL by TMIG-IADL at 6 Months After Surgery

Factor	Classification	Number of Patients	Rate of No Deterioration (95% CI)	OR (95% CI)	*p* Value
Sex	Male	491	82.7 (79.1–85.9)	1 (reference)	
	Female	385	88.1 (84.4–91.1)	0.648 (0.440–0.954)	0.0280
Age (y)	≤79	504	87.7 (84.5–90.4)	1 (reference)	
	≥80	372	81.5 (77.1–85.3)	1.624 (1.118–2.357)	0.0108
Age (y)	≤84	805	86.0 (83.4–88.3)	1 (reference)	
	≥85	71	74.7 (62.9–84.2)	2.080 (1.176–3.679)	0.0119
Clinical stage	0	29	86.2 (68.3–96.1)	1 (reference)	
	IA1	131	91.6 (85.5–95.7)	0.573 (0.169–1.946)	0.3720
	IA2	254	87.4 (82.7–91.2)	0.901 (0.294 – 2.757)	0.8549
	IA3	158	87.3 (81.1–92.1)	0.906 (0.285–2.875)	0.8667
	IB	134	79.9 (72.1–86.3)	1.577 (0.506–4.915)	0.4321
	IIA	36	94.4 (81.3–99.3)	0.368 (0.062–2.169)	0.2693
	IIB	96	74.0 (64.0–82.4)	2.201 (0.697–6.948)	0.1787
	IIIA+B	38	73.7 (56.9–86.6)	2.232 (0.621–8.019)	0.2184
ECOG Performance status	0	717	87.6 (85.0–89.9)	1 (reference)	
	1	152	73.7 (65.9–80.5)	2.520 (1.650–3.850)	<0.0001
	2–4	7	71.4 (29.0–96.3)	2.823 (0.539–14.767)	0.2191
Smoking history	Never	363	88.7 (85.0–91.8)	1 (reference)	
	Ever	513	82.5 (78.9–85.7)	1.671 (1.124–2.485)	0.0112
Smoking years	0	364	88.7 (85.0–91.8)	1 (reference)	
	1–19	51	94.1 (83.8–98.8)	0.493 (0.147–1.653)	0.2518
	20–39	132	78.8 (70.8–85.4)	2.121 (1.250–3.599)	0.0053
	≥40	328	82.0 (77.4–86.0)	1.728 (1.124–2.656)	0.0127
Respiratory comorbidity	No	588	87.9 (85.0–90.5)	1 (reference)	
	Yes	288	79.2 (74.0–83.7)	1.916 (1.314–2.795)	0.0007
Emphysema	No	623	87.3 (84.5–89.8)	1 (reference)	
	Yes	253	79.5 (73.9–84.3)	1.781 (1.211–2.620)	0.0033
Interstitial pneumonia	No	788	86.3 (83.7–88.6)	1 (reference)	
	Yes	88	73.9 (63.4–82.7)	2.228 (1.328–3.736)	0.0024
Number of medications	0	63	92.1 (82.4–97.4)	1 (reference)	
	1-3	255	89.8 (85.4–93.2)	1.317 (0.458–3.578)	0.5893
	4-9	464	83.0 (79.2–86.3)	2.380 (0.925–6.124)	0.0721
	≥10	94	77.7 (67.9–85.6)	3.337 (1.186–9.388)	0.0224
Operation	Wedge Resection	95	88.4 (80.2–94.1)	1 (reference)	
	Segmentectomy	133	81.2 (73.5–87.5)	1.768 (0.823–3.796)	0.1441
	Lobectomy	639	85.5 (82.5–88.1)	1.301 (0.668–2.531)	0.4390
	Bilobectomy	6	83.3 (35.9–99.6)	1.527 (0.163–14.305)	0.7106
	Pneumonectomy	3	66.7 (9.4–99.2)	3.818 (0.319–46.657)	0.2899
Lymph node dissection	ND0–1	371	84.6 (80.6–88.2)	1 (reference)	
	≥ND2a	505	85.4 (82.0–88.3)	0.946 (0.650–1.376)	0.7708
Combined resection	No	863	85.4 (82.9–87.7)	1 (reference)	
	Yes	13	61.5 (31.6–86.1)	3.656 (1.177–11.353)	0.0250
Operation time	<180 min	548	83.8 (80.4–86.8)	1 (reference)	
	≥180 min	328	87.2 (83.1–90.6)	0.757 (0.510–1.125)	0.1685
G8 score at registration	≥15	380	90.5 (87.1–93.3)	1 (reference)	
	≤14	495	80.8 (77.1–84.2)	2.269 (1.506–3.149)	<0.0001
CCI score	0	447	88.8 (85.5–91.6)	1 (reference)	
	≥1	429	81.1 (77.1–84.7)	1.848 (1.263–2.704)	0.0016

ADL, activities of daily living; CCI, Charlson comorbidity index; CI, confidence interval; ECOG, Eastern Cooperative Oncology Group; G8, geriatric screening tool; ND0, no node dissection; ND1, hilar node dissection; ND2, mediastinal node dissection; TMIG-IADL, Tokyo Metropolitan Institute of Gerontology Index of Competence Instrumental Activities of Daily Living.

Multivariable analysis indicated that only the following four clinical factors were associated with significant ADL deterioration, namely: (1) poor preoperative PS; (2) low G8 score; (3) segmentectomy (versus wedge resection); and (4) surgery lasting shorter than 3 hours (see Supplementary Table 1*F* for results of the multivariable analysis, which details the four significant factors).

### Changes in Specific ADL Subscales

Among the 841 patients with available data on TMIG-IADL scores at 6 months, a score worsening of at least one point was present in the instrumental self-maintenance, effectance, and social role domains in 16.2%, 17.0%, and 36.4% of the patients, respectively (Table 3). The worsening seemed to be strongest in the social role domain. We considered that this finding might have reflected the coronavirus disease 2019 (COVID-19) pandemic, and performed additional analyses in patients categorized according to the time of enrollment.

**Table 3:** Worsening of Scores in Each Specific Item and Domain in the Tokyo Metropolitan Institute of Gerontology Index of Competence Instrumental Activities of Daily Living Among Patients Who Responded to Both the Preoperative and Postoperative Surveys (N = 841)

Item	Number of Patients Reporting Score Worsening	% (95% CI)
1. Can you use public transportation (bus or train) by yourself?	73	8.7 (6.9–10.8)
2. Are you able to shop for daily necessities?	42	5.0 (3.6–6.7)
3. Are you able to prepare meals by yourself?	66	7.9 (6.1–9.9)
4. Are you able to pay bills?	37	4.4 (3.1–6.0)
5. Can you handle your own banking?	43	5.1 (3.7–6.8)
Domain: Instrumental self-maintenance (items 1–5) by one point or more	136	16.2 (13.8–18.8)
Domain: Instrumental self-maintenance (items 1–5) by two points or more	60	7.1 (5.5–9.1)
6. Are you able to fill out forms for your pension?	58	6.9 (5.3–8.8)
7. Do you read newspapers?	44	5.2 (3.8–7.0)
8. Do you read books or magazines?	66	7.9 (6.1–9.9)
9. Are you interested in news stories or programs dealing with health?	31	3.7 (2.5–5.2)
Domain: Effectance (items 6–9) by one point or more	143	17.0 (14.5–19.7)
Domain: Effectance (items 6–9) by two points or more	37	4.4 (3.1–6.0)
10. Do you visit the homes of friends?	135	16.1 (13.6–18.7)
11. Are you sometimes called on for advice?	113	13.4 (11.2–15.9)
12. Are you able to visit sick friends?	142	16.9 (14.4–19.6)
13. Do you sometimes initiate conversations with young people?	87	10.3 (8.4–12.6)
Domain: Social role (items 10–13) by one point or more	306	36.4 (33.1–39.7)
Domain: Social role (items 10–13) by two points or more	120	14.3 (12.0–16.8)

CI, confidence interval.

Among the 230 patients enrolled before September 2019 (for whom the primary end point was evaluated before April 2020, when the first state of emergency for the COVID-19 pandemic was declared in Japan), a score worsening of greater than or equal to one point at 6 months was detected in the instrumental self-maintenance, effectance, and social role domains in 12.6%, 17.8%, and 30.9% of the patients, respectively. Conversely, a score worsening of greater than or equal to one point at 6 months was detected in the instrumental self-maintenance, effectance, and social role domains in 17.5%, 16.7%, and 38.5% of the 611 patients enrolled after October 2019 (Supplementary Table 1*G*). The differences between pre and post–COVID-19 score changes were not statistically significant except for social role domain score (*p* = 0.0426 by chi-square test). Therefore, despite the presence of a certain degree of COVID-19 effect on ADL, this effect was unlikely to be great and the “social role” was the most affected domain in both periods.

### Changes in QOL

The preoperative and postoperative EQ-5D-5L questionnaire data were available for 864 (98.6%) and 821 (93.7%) of the 876 patients, respectively. The score changes could be analyzed in 811 patients (92.6%) who were also included in the primary end point analysis. On the basis of an MID of 0.061 for the EQ-5D-5L score, QOL deterioration (by MID or more) was observed in 179 patients (22.1%), whereas 115 (14.2%) reported QOL worsening by less than MID, 280 (34.5%) reported no change, 85 (10.5%) reported QOL improvement by less than MID, and 152 (18.7%) reported improvement by MID or more. Preoperative poor PS (2–4 versus 0) and smoking history (ever versus never) were significantly correlated with predetermined (i.e., by MID or more) QOL deterioration at 6 months. QOL score changes were not significantly different between those with and without grade 3 or higher serious postoperative complications, although this comparison is biased because they were limited to patients “fit enough” to fill in postoperative QOL questionnaires.

**Figure 3:**
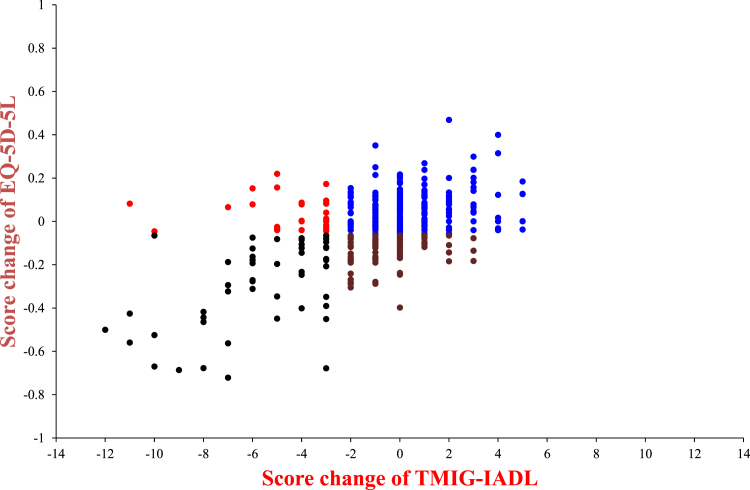
Correlation between changes in the TMIG-IADL score and the changes in the EQ-5D-5L score after surgery. Red and black dots represent those with substantial ADL deterioration (TMIG-IADL score worsened by ≥3 points), whereas brown and black dots represent those with substantial QOL deterioration (EQ-5D-5L score worsened by ≥0.061 points). TMIG-IADL and EQ-5D-5L scores were poorly correlated (Spearman’s rank correlation coefficient = 0.2884). EQ-5D-5L, EuroQol 5-dimensions 5-level; QOL, quality of life; TMIG-IADL, Tokyo Metropolitan Institute of Gerontology Index of Competence Instrumental Activities of Daily Living.

The correlation analysis including these 811 patients indicated that the changes in TMIG-IADL and EQ-5D-5L scores were poorly correlated (Spearman’s rank correlation coefficient = 0.2884), as illustrated in Figure 3. The kappa coefficient for the agreement between deterioration/nondeterioration of TMIG-IADL (by ≥3 points) and QOL (by MID of ≥0.061) scores was 0.2655, confirming poor correlation (see Supplementary Table 1*H* for specific data).

Of the 811 patients with both baseline and postoperative EQ-5D-5L scores, 125 (15.4%) and 154 (19.0%) revealed improvement in Pain/Discomfort item and Anxiety/Depression item, respectively. These patients were more likely to report improved TMIG-IADL or overall QOL scores (Supplementary Table 1*I*).

## Discussion

In the present study, data on preoperative and postoperative ADL could be obtained from 98.1% of 876 elderly patients undergoing curative surgery for NSCLC who were alive at 6 months. In nine of the 16 alive patients with missing data, the cause of their exclusion was incapacitating complications, which were undoubtedly associated with ADL deterioration as we had anticipated when determining the primary end point of the study. Therefore, data on ADL were only “truly missing” from seven of the 876 patients (<1%). In addition, data on QOL could be obtained from greater than 90% of the patients. Therefore, in the current study, evaluating postoperative ADL and QOL outcomes was feasible despite the disruptive effect of the COVID-19 pandemic during the study period.

With the aging population in Japan, the number of elderly patients with lung cancer, including those undergoing surgical resection, is rapidly increasing.^1^^,^^3^ Despite improvements in surgical safety and OS,^2^^,^^3^ data on the impact of surgery on long-term ADL and QOL, which are critically important for patients and families, are limited.^8^^,^^10^^,^^11^ Previous studies have focused on short-term morbidity and mortality of surgical procedures and OS.^2^, ^3^, ^4^ However, aside from survival, the ADL and QOL of patients as cancer survivors should also be evaluated.

In the present study, we confirmed the favorable survival outcomes of surgery in elderly patients with NSCLC, on the basis of surgical mortality and 6-month OS rates of 0.3% and 98.7%, respectively. However, although there was considerable variability in postoperative ADL change, with some suffering from remarkable worsening and others reporting maintained or even improved activities, ADL scores significantly tended to worsen at postoperative 6 months. Protocol-specified ADL deterioration (defined as a decline of ≥3 points in TMIG-IADL score or missing ADL data) was not observed in only 85% of the patients, indicating that the remaining 15% of the patients experienced significant functional loss after surgery.

ADL maintenance or recovery was not determined solely by short-term postoperative complications. Although patients who experienced grade 3 or higher surgical complications were statistically significantly more likely to suffer from ADL deterioration, more than 60% achieved ADL recovery at 6 months, with some even reporting “improved” postoperative ADL or QOL. On the other hand, one out of every eight patients without grade 3 or higher postoperative complications experienced significant ADL deterioration. Therefore, operative complications are not a surrogate for long-term functional recovery, and ADL should be evaluated as a distinct end point.

Univariable analyses revealed that male sex, age of at least 80 years, poor PS, smoking history, emphysema, interstitial pneumonia, daily use of multiple medications, low baseline G8 score, high CCI score, and combined resection were associated with postoperative ADL deterioration at 6 months. In multivariable analysis, the following four predictive factors remained significantly associated with ADL deterioration at 6 months: (1) poor preoperative PS; (2) low baseline G8 score; (3) segmentectomy (versus wedge resection); and (4) surgery lasting shorter than 3 hours. The clinical relevance of the observed association between shorter surgery duration and ADL deterioration remains unclear and could be a statistical anomaly. Or it might be owing to bias that clinically higher-risk patients underwent operation by more experienced surgeons. In any case, it could be concluded that having shorter surgical time would not lead to the maintenance of postoperative ADL. Information on these predictive factors would aid in patient counseling, treatment decision-making, and perioperative care for elderly patients.

Although there were no significant correlations between surgical procedures and ADL deterioration by univariable comparisons, the multivariable analysis did indicate that wedge resection was associated with less ADL deterioration, especially versus segmentectomy. In a recently published Cancer and Leukemia Group B trial,^29^ “sublobar” resection was found to be noninferior to lobectomy in early-stage NSCLC, which is compatible with the study by Japan Clinical Oncology Group.^15^ However, in this Cancer and Leukemia Group B trial, both wedge resection and segmentectomy were included in the “sublobar resection” group. Given the different effects on ADL, it might be inappropriate to put wedge resection and segmentectomy together as “less invasive” surgical procedures.

Of note, preoperative comprehensive geriatric assessments were significantly associated with short-term surgical complications during the first 30 days in the previous study by the Japanese Association for Chest Surgery.^3^ In the present study, we used the simplified G8 assessment,^22^ which was also associated with functional outcomes at a longer term of 6 months. On the other hand, age alone did not predict functional outcomes. These results reconfirm the importance of pretreatment assessment using geriatric scales.^3^^,^^30^^,^^31^ The currently ongoing data collection and analyses aim to determine whether patients experience additional ADL deterioration during longer follow-up periods of 12 and 24 months after surgery and to identify predictive factors.

We also collected QOL data using the EQ-5D-5L questionnaire, given that QOL and ADL are not identical. In fact, in the current study, not only the postoperative data retrieval rates were different, but the changes in TMIG-IADL and EQ-5D-5L scores were poorly correlated. QOL deterioration by the MID or more was reported by 22.1% of the patients, which was higher than the rate of patients with ADL deterioration. These results could have reflected the different data collection methods but suggest that ADL and QOL should be separately evaluated despite their comparable importance. Future studies should elucidate whether ADL or QOL has a bigger impact on long-term outcomes in elderly patients undergoing surgery for lung cancer.

The present study has several strengths. This was a multi-institutional prospective study with large cohort size. Previous studies on postoperative patient-reported outcomes (PROs) reported only modest responses. For example, in a European study on the economic burden of patients with resected NSCLC,^32^ the authors reported that 306 of the 526 (58%) invited patients completed the survey. In another study by Heiden et al., ^33^ which investigated the PROs after NSCLC resection in 334 patients, each of the PRO scores at 6 months could be collected from only half the patients or less. In our study, however, the PRO data retrieval rates were very high for both ADL and QOL, despite the disruptive effect of the COVID-19 pandemic on clinical trials.^34^

On the other hand, we also acknowledge the weaknesses and the limitations of the study. First, as is the case in most surgical observational studies, our patients are selected for curative operation and are undoubtedly “fitter” than the general elderly population. Most of them have a PS of 0, with few comorbidities, and the SD of baseline ADL was smaller. The generalizability of our results to more frail populations, thus, remains unclear. Second, although the cutoff value for the QOL has been set at 0.061 according to a previous report on MID,^28^ no data are available regarding the changes in the TMIG-IADL score for clinically relevant ADL deterioration. We embraced the distribution-based method^27^ and defined deterioration as a change of one SD in the TMIG-IADL score. Although used in previous studies evaluating the effect of brain radiotherapy cognitive functions,^35^, ^36^, ^37^ this approach could be criticized as arbitrary.

Third, we did not have reference data to determine whether the rate of patients experiencing ADL deterioration of 15% was expected or not. Puts et al.^38^ investigated the 6 months postoperative ADL in 112 elderly patients who underwent breast cancer surgery and reported that 21.9% suffered from functioning deterioration, with which no variable was associated. In a larger, the international, multicenter Geriatric Oncology Surgical Assessment and Functional rEcovery after Surgery (GOSAFE) study,^39^ 945 elderly patients who underwent surgery for various cancers were analyzed for EQ-5D, with worsening and recovered scores at postoperative 3 and 6 months, respectively. No definitions of MID were given in either report. In a study with longer (5-year) follow-up, ADL change was investigated in 239 elderly patients with breast cancer, and Lemij et al.^40^ reported that treatment was not associated with physical activities. Although it seemed that our results are not inconsistent with these previous reports, direct comparisons are difficult owing to differences in disease status and evaluation methods. In particular, we cannot precisely differentiate the effect of surgery-induced versus “normal” deterioration process of the aged patients, although it would be unlikely that many “normal” elderly people suffer from a significant functional loss in 6 months. Comparisons with other modalities, such as radiotherapy, would be greatly informative for treatment selection. Fourth, information on the excluded patients, such as those who underwent noncurative surgery, is missing. In fact, some of the patients might have received effective target-based drugs with no ADL deterioration.

Future oncology trials should adopt a comprehensive approach and include ADL and QOL assessment irrespective of the treatment modalities chosen. There was a significant number of patients who reported improvement of postoperative ADL or QOL; 185 (21.1%) reported TMIG-IADL score improvement, and 152 (18.7%) reported that their QOL was improved by MID or more. This could be attributed to the physical and psychological burden of cancer and the subsequent curative operation leading to “disease-free” status.

In conclusion, our analyses of ADL and QOL in a large cohort of patients from a large number of institutions across Japan revealed that, even in this selected group with limited generalizability, a significant proportion of elderly patients who underwent curative surgical resection for NSCLC experienced ADL deterioration at 6 months after surgery, highlighting the need for further studies elucidating the predictive and contributory factors. The fact that ADL and QOL changes were not uniform, with some reporting improved postoperative ADL/QOL, makes it all the more necessary to have better tools in predicting who will feel better versus worse after surgery.

## CRediT Authorship Contribution Statement

**Hidefumi Takei:** Conceptualization, Investigation, Methodology, Resources, Visualization, Writing - original draft, Writing - review & editing.

**Hideo Kunitoh:** Conceptualization, Funding acquisition, Methodology, Project administration, Supervision, Visualization, Writing - original draft, Writing - review & editing.

**Masashi Wakabayashi:** Data curation, Formal analysis, Methodology, Software, Validation, Visualization, Writing - review & editing.

**Tomoko Kataoka:** Data curation, Software, Validation, Writing - review & editing.

**Yuta Sekino:** Data curation, Software, Validation, Writing - review & editing.

**Tomonori Mizutani:** Conceptualization, Methodology, Visualization, Writing - review & editing.

**Masahiro Tsuboi:** Investigation, Resources, Writing - review & editing.

**Norihiko Ikeda:** Investigation, Resources, Writing - review & editing.

**Hisao Asamura:** Investigation, Resources, Writing - review & editing.

**Morihito Okada:** Investigation, Resources, Writing - review & editing.

**Makoto Takahama:** Investigation, Resources, Writing - review & editing.

**Yasuhisa Ohde:** Investigation, Resources, Writing - review & editing.

**Jiro Okami:** Investigation, Resources, Writing - review & editing.

**Satoshi Shiono:** Investigation, Resources, Writing - review & editing.

**Keijyu Aokage:** Investigation, Project administration, Resources, Writing - review & editing.

**Shun-ichi Watanabe:** Conceptualization, Funding acquisition, Project administration, Supervision, Writing - original draft, Writing - review & editing.
