# Supplementary material for: Prospective, Multi-Institutional Observational Study of Deterioration in Activities of Daily Living in Elderly Patients After Lung Cancer Surgery
Source: JTO Clin Res Rep. 2023 Jul 17;4(8):100550. doi: 10.1016/j.jtocrr.2023.100550 (PMC10432798; doi:10.1016/j.jtocrr.2023.100550)
Supplement: Supplementary Table 1 [file mmc1.docx]

**Appendix: Supplemental Data**

Table A1. Tokyo Metropolitan Institute of Gerontology Index of Competence Instrumental Activities of Daily Living questionnaire

| Domain | Item | Score |
| --- | --- | --- |
| Instrumental Self-maintenance | Can you use public transportation (bus or train) by yourself? | Yes: 1 No: 0 |
|  | Are you able to shop for daily necessities? | Yes: 1 No: 0 |
|  | Are you able to prepare meals by yourself? | Yes: 1 No: 0 |
|  | Are you able to pay bills? | Yes: 1 No: 0 |
|  | Can you handle your own banking? | Yes: 1 No: 0 |
| Effectance | Are you able to fill out forms for your pension? | Yes: 1 No: 0 |
|  | Do you read newspapers? | Yes: 1 No: 0 |
|  | Do you read books or magazines? | Yes: 1 No: 0 |
|  | Are you interested in news stories or programs dealing with health? | Yes: 1 No: 0 |
| Social role | Do you visit the homes of friends? | Yes: 1 No: 0 |
|  | Are you sometimes called on for advice? | Yes: 1 No: 0 |
|  | Are you able to visit sick friends? | Yes: 1 No: 0 |
|  | Do you sometimes initiate conversations with young people? | Yes: 1 No: 0 |

Table A2. G8 geriatric screening questionnaire

| Item | Score |
| --- | --- |
| 1. Has food intake declined over the past 3 months due to loss of appetite, digestive problems, chewing, or swallowing difficulties? | 0 = severe reduction in food intake  1 = moderate reduction in food intake |
|  | 2 = normal food intake |
| 2. Weight loss during the last 3 months | 0 = weight loss > 3 kg |
|  | 1 = does not know |
|  | 2 = weight loss between 1 and 3 kg |
|  | 3 = no weight loss |
| 3. Mobility | 0 = bed- or chair-bound |
|  | 1 = able to get out of bed/chair but does not go out |
|  | 2 = goes out |
| 4. Neuropsychological problems | 0 = severe dementia or depression |
|  | 1 = mild dementia or depression |
|  | 2 = no psychological problems |
| 5. Body mass index (kg/m^2^) | 0 = body mass index <19 |
|  | 1 = 19 < body mass index <21 |
|  | 2 = 21 < body mass index <23 |
|  | 3 = body mass index > 23 |
| 6. Takes more than three medications per day | 0 = yes |
|  | 1 = no |
| 7. In comparison with other people of the same age, how does the patient consider his/her health status? | 0.0 = not as good  0.5 = does not know |
|  | 1.0 = as good |
|  | 1.5 = better |
| 8. Age | 0 = > 85 years |
|  | 1 = 80-85 years |
|  | 2 = < 80 years |

Table A3. Charlson comorbidity index

| Conditions | Assigned weight for diseases |
| --- | --- |
| Myocardial infarct | 1 |
| Congestive heart failure | 1 |
| Peripheral vascular disease | 1 |
| Cerebrovascular disease | 1 |
| Dementia | 1 |
| Chronic pulmonary disease | 1 |
| Connective tissue disease | 1 |
| Peptic ulcer disease | 1 |
| Mild liver disease | 1 |
| Diabetes | 1 |
| Hemiplegia | 2 |
| Moderate or severe renal disease | 2 |
| Diabetes with end organ damage | 2 |
| Solid tumor without metastasis for 5 years | 2 |
| Leukemia | 2 |
| Lymphoma | 2 |
| Moderate or severe liver disease | 3 |
| Metastatic solid tumor | 6 |
| Acquired immunodeficiency syndrome | 6 |

Table A4. EuroQol 5-dimentions 5-level questionnaire

| Item | Status |
| --- | --- |
| MOBILITY | 1. I have no problems in walking about |
|  | 1. I have slight problems in walking about |
|  | 1. I have moderate problems in walking about |
|  | 1. I have severe problems in walking about |
|  | 1. I am unable to walk about |
| SELF-CARE | 1. I have no problems washing or dressing myself |
|  | 1. I have slight problems washing or dressing myself |
|  | 1. I have moderate problems washing or dressing myself |
|  | 1. I have severe problems washing or dressing myself |
|  | 1. I am unable to wash or dress myself |
| USUAL ACTIVITIES | 1. I have no problems doing my usual activities |
|  | 1. I have slight problems doing my usual activities |
|  | 1. I have moderate problems doing my usual activities |
|  | 1. I have severe problems doing my usual activities |
|  | 1. I am unable to do my usual activities |
| PAIN/DISCOMFORT | 1. I have no pain or discomfort |
|  | 1. I have slight pain or discomfort |
|  | 1. I have moderate pain or discomfort |
|  | 1. I have severe pain or discomfort |
|  | 1. I have extreme pain or discomfort |
| ANXIETY/DEPRESSION | 1. I am not anxious or depressed |
|  | 1. I am slightly anxious or depressed |
|  | 1. I am moderately anxious or depressed |
|  | 1. I am severely anxious or depressed |
|  | 1. I am extremely anxious or depressed |

Table A5. Classification of clinical and pathological factors for univariable subset analyses and multivariable logistic regression

| Factor | Classifications for univariate subset analysis | Classifications for multivariable logistic regression |
| --- | --- | --- |
| Sex | male vs female | male vs female |
| Age (years) | <79 vs >80; <84 vs >85 | <79 vs >80 |
| Clinical stage | 0 vs 1A1; 0 vs 1A2; 0 vs 1A3; 0 vs 1B; 0 vs IIA; 0 vs IIB; 0 vs IIIA–B | 0 vs 1A1–1B; 0 vs IIA–IIIB |
| Performance status | 0 vs 1; 0 vs 2–4 | 0 vs 1–4 |
| Smoking history | never vs ever |  |
| Smoking years | 0 vs 1–19; 0 vs 20–39; 0 vs >40 | 0 vs 1–19; 0 vs >20 |
| Respiratory comorbidity | no vs yes |  |
| Emphysema | no vs yes | no vs yes |
| Interstitial pneumonia | no vs yes | no vs yes |
| Number of medications | 0 vs 1–3; 0 vs 4–9; 0 vs >10 | 0–3 vs >4 |
| Operation | WED vs SEG; WED vs LOB; WED vs BiLOB; WED vs PNE | WED vs SEG; WED vs (LOB or BiLOB or PNE) |
| Lymph node dissection | ND0–1 vs > ND2a | ND0–1 vs > ND2a |
| Combined resection | no vs yes | no vs yes |
| Operation time (min) | <180 vs >180 | <180 vs >180 |
| G8 score at registration | >15 vs <14 | >15 vs <14 |
| CCI score | 0 vs >1 | 0 vs >1 |

WED, wedge resection; SEG, segmentectomy; LOB, lobectomy; BiLOB, bilobectomy; PNE, pneumonectomy; ND0, no node dissection; ND1, hilar node dissection; ND2, mediastinal node dissection; CCI, Charlson comorbidity index

Table A6. Multivariable analysis of factors associated with non-deterioration of ADL by TMIG-IADL at 6 months after surgery

| Factor | Classification | Number of patients | %non-deterioration rate (95% CI) | Odds ratio (95% CI) | p value |
| --- | --- | --- | --- | --- | --- |
| Sex | Male | 491 | 82.7 (79.1–85.9) | 1 (Reference) |  |
|  | Female | 385 | 88.1 (84.4–91.1) | 0.928 (0.510–1.687) | 0.8061 |
| Age (years) | <79 | 504 | 87.7 (84.5–90.4) | 1 (Reference) |  |
|  | >80 | 372 | 81.5 (77.1–85.3) | 1.264 (0.826–1.932) | 0.2802 |
| Clinical stage | 0 | 29 | 86.2 (68.3–96.1) | 1 (Reference) |  |
|  | IA1-IB | 677 | 86.7 (83.9–89.2) | 0.900 (0.280–2.890) | 0.8952 |
|  | IIA-IIIB | 170 | 78.2 (71.3–84.2) | 1.390 (0.394–4.905) | 0.6088 |
| ECOG PS | 0 | 717 | 87.6 (85.0–89.9) | 1 (Reference) |  |
|  | 1-4 | 159 | 73.6 (66.0–80.3) | 2.040 (1.296–3.212) | 0.0021 |
| Smoking history | Never | 363 | 88.7 (85.0–91.8) | 1 (Reference) |  |
|  | <20 years | 52 | 94.2 (84.1–98.8) | 0.446 (0.125–1.586) | 0.2121 |
|  | >20 years | 460 | 81.1 (77.2–84.6) | 1.420 (0.741–2.721) | 0.2913 |
| Emphysema | No | 623 | 87.3 (84.5–89.8) | 1 (Reference) |  |
|  | Yes | 253 | 79.5 (73.9–84.3) | 1.204 (0.737–1.967) | 0.4593 |
| Interstitial pneumonia | No | 788 | 86.3 (83.7–88.6) | 1 (Reference) |  |
|  | Yes | 88 | 73.9 (63.4–82.7) | 1.766 (0.987–3.161) | 0.0554 |
| Number of medications | <3 | 318 | 90.3 (86.5–93.3) | 1 (Reference) |  |
|  | >4 | 558 | 82.1 (78.6–85.2) | 1.395 (0.875–2.224) | 0.1618 |
| Operation | Wedge resection | 95 | 88.4 (80.2–94.1) | 1 (Reference) |  |
|  | Segmentectomy | 133 | 81.2 (73.5–87.5) | 3.308 (1.443–7.585) | 0.0047 |
|  | Lobectomy or more | 648 | 85.3 (82.4–88.0) | 2.214 (0.993–4.936) | 0.0520 |
| Lymph node dissection | ND0–1 | 371 | 84.6 (80.6–88.2) | 1 (Reference) |  |
|  | >ND2a | 505 | 85.4 (82.0–88.3) | 0.985(0.603 – 1.606) | 0.9502 |
| Combined resection | No | 863 | 85.4 (82.9–87.7) | 1 (Reference) |  |
|  | Yes | 13 | 61.5 (31.6–86.1) | 2.187(0.633 – 7.559) | 0.2164 |
| Operation time (min) | <180 min | 548 | 83.8 (80.4–86.8) | 1 (Reference) |  |
|  | >180 min | 328 | 87.2 (83.1–90.6) | 0.598 (0.386–0.929) | 0.0220 |
| G8 score at registration | >15 | 380 | 90.5 (87.1–93.3) | 1 (Reference) |  |
|  | <14 | 495 | 80.8 (77.1–84.2) | 1.864 (1.172–2.964) | 0.0085 |
| CCI score | 0 | 447 | 88.8 (85.5–91.6) | 1 (Reference) |  |
|  | >1 | 429 | 81.1 (77.1–84.7) | 1.192 (0.775–1.834) | 0.4239 |

ADL, activities of daily living; TMIG-IADL, Tokyo Metropolitan Institute of Gerontology Index of Competence Instrumental Activities of Daily Living; CI, confidence interval; ECOG PS, Eastern Cooperative Oncology Group performance status; ND0, no node dissection; ND1, hilar node dissection; ND2, mediastinal node dissection; Charlson comorbidity index

Table A7. Deterioration of scores in each specific item and domain in the Tokyo Metropolitan Institute of Gerontology Index of Competence Instrumental Activities of Daily Living according to the enrollment period during the COVID-19 era

| Item | Number (%) of patients reporting score worsening accrued before September, 2019 (N = 230) | Number (%) of patients reporting score worsening accrued after October, 2019 (N = 611) |
| --- | --- | --- |
| 1 Can you use public transportation (bus or train) by yourself? | 15 (6.5%) | 58 (9.5%) |
| 2 Are you able to shop for daily necessities? | 5 (2.2%) | 37 (6.1%) |
| 3 Are you able to prepare meals by yourself? | 13 (5.7%) | 53 (8.7%) |
| 4 Are you able to pay bills? | 9 (3.9%) | 28 (4.6%) |
| 5 Can you handle your own banking? | 9 (3.9%) | 34 (5.6%) |
| Domain: Instrumental self–maintenance (items 1–5) by 1 point or more | 29 (12.6%) | 107 (17.5%) |
| Domain: Instrumental self–maintenance (items 1–5) by 2 points or more | 12 (5.2%) | 48 (7.9%) |
| 6 Are you able to fill out forms for your pension? | 15 (6.5%) | 43 (7.0%) |
| 7 Do you read newspapers? | 10 (4.4%) | 34 (5.6%) |
| 8 Do you read books or magazines? | 22 (9.6%) | 44 (7.2%) |
| 9 Are you interested in news stories or programs dealing with health? | 10 (4.4%) | 21 (3.4%) |
| Domain: Effectance (items 6–9) by 1 point or more | 41 (17.8%) | 102 (16.7%) |
| Domain: Effectance (items 6–9) by 2 points or more | 12 (5.2%) | 25 (4.1%) |
| 10 Do you visit the homes of friends? | 27 (11.7%) | 108 (17.7%) |
| 11 Are you sometimes called on for advice? | 25 (10.9%) | 88 (14.4%) |
| 12 Are you able to visit sick friends? | 28 (12.2%) | 114 (18.7%) |
| 13 Do you sometimes initiate conversations with young people? | 24 (10.9%) | 63 (10.3%) |
| Domain: Social role (items 10–13) by 1 point or more | 71 (30.9%) | 235 (38.5%) |
| Domain: Social role (items 10–13) by 2 points or more | 23 (10.0%) | 97 (15.9%) |

Table A8. Correlation between ADL deterioration determined using the TMIG-IADL questionnaire and QOL deterioration using the EQ-5D-5L questionnaire at 6 months after surgery.

|  | No QOL deterioration | QOL deterioration | Total |
| --- | --- | --- | --- |
| No ADL deterioration | 591 | 128 | 719 |
| ADL deterioration | 41 | 51 | 92 |
| Total | 632 | 179 | 811 |

Kappa coefficient, 0.2665 (95% CI: 0.1876–0.3453)

ADL, activities of daily living; TMIG-IADL, Tokyo Metropolitan Institute of Gerontology Index of Competence Instrumental Activities of Daily Living; QOL, quality of life; EQ-5D-5L, EuroQol 5-dimentions 5-level; CI, confidence interval

Table A9. ADL change by the TMIG-IADL questionnaire and QOL change by the EQ-5D-5L questionnaire, according to the EQ-5D-5L PAIN/DISCOMFORT and ANXIETY/DEPRESSION score change (N=811).

| Item | Change | N | ADL worsened | ADL stable | ADL improved | QOL worsened | QOL stable | QOL improved |
| --- | --- | --- | --- | --- | --- | --- | --- | --- |
| P/D | better | 125 | 41 (32.8%) | 46 (36.8%) | 38 (30.4%) | 23 (18.4%) | 0 (0) | 102 (81.6%) |
| P/D | no change | 485 | 152 (31.3%) | 223 (46.0%) | 110 (22.7%) | 101 (20.8%) | 280 (57.7%) | 104 (21.4%) |
| P/D | worse | 201 | 101 (50.2%) | 72 (35.8%) | 28 (13.9%) | 170 (84.6%) | 0 (0) | 31 (15.4%) |
| A/D | better | 154 | 57 (37.0%) | 56 (36.4%) | 41 (26.6%) | 28 (18.2%) | 0 (0) | 126 (81.8%) |
| A/D | no change | 570 | 184 (32.3%) | 264 (46.3%) | 122 (21.4%) | 184 (32.3%) | 280 (49.1%) | 106 (18.6%) |
| A/D | worse | 87 | 53 (60.9%) | 21 (24.1%) | 13 (14.9%) | 82 (94.3%) | 0 (0) | 5 (5.7%) |
|  |  |  |  |  |  |  |  |  |

ADL, activities of daily living; TMIG-IADL, Tokyo Metropolitan Institute of Gerontology Index of Competence Instrumental Activities of Daily Living; QOL, quality of life; EQ-5D-5L, EuroQol 5-dimentions 5-level; P/D, PAIN/DISCOMFORT; A/D, ANXIETY/DEPRESSION
